# Plain Language Summary

**DOI:** 10.2903/j.efsa.2023.p210901

**Published:** 2023-09-13

## Abstract

This publication is linked to the following EFSA Journal article: https://efsa.onlinelibrary.wiley.com/doi/10.2903/j.efsa.2023.8215

efsa JOURNAL

13 September 2023

## Update of the risk assessment of mineral oil hydrocarbons (MOH) in food

### Background

- Mineral oil hydrocarbons (MOH) are substances derived primarily from crude oil, but also from coal, gas and biomass. There are two main classifications of MOH: mineral oil-saturated hydrocarbons (MOSH) and mineral oil aromatic hydrocarbons (MOAH).
- MOH can enter food as environmental contaminants, lubricants from machinery used during harvesting and food production, processing aids like release agents or dust binders, food or feed additives, and food contact materials. MOH products that are used in food are treated to minimise the MOAH content.
- In 2012, the Scientific Panel on Contaminants in the Food Chain (CONTAM Panel) of the European Food Safety Authority (EFSA) concluded that the potential human health impact of MOH varies widely depending on the MOH type. Additionally, the CONTAM Panel concluded in 2012 that MOSH can accumulate in human tissue and can harm the liver, while MOAH, especially those with chemical structures including 3–7 aromatic rings may cause cancer by damaging DNA. Due to insufficient analytical data, the European Commission (EC) has organised the monitoring of MOH in food and all food contact surfaces.

### Why is EFSA writing about MOH?

The EC is considering whether to establish maximum levels for MOH in specific food items. To inform this decision, the EC mandated EFSA in 2020 to update the CONTAM Panel Scientific Opinion from 2012 on the risks to public health from MOH in food.

### What was EFSA asked to do?

EFSA was asked to:

- Review research on MOH toxicity since 2012 and update the scientific opinion on the hazard characterisation (potential for harm) of MOH.
- Estimate the exposure levels of MOH considering recent occurrence data; and consequently, update the risk characterisation (the likelihood that harm will happen) of MOH, based on these recent data.

### How did EFSA carry out this work?

- For the assessment of MOH, EFSA has strictly adhered to a pre-established methodology and documented the entire process.
- The draft scientific opinion based on the reassessment underwent a public consultation from 15 March to 30 April 2023.

### What data were used?

- EFSA completed a comprehensive review of the scientific literature on various MOH topics, including the identification of toxicokinetics and toxicity data, focusing on studies published since 2010. The first literature search was made between 16 January and 5 February 2021. A second search was performed on 10 October 2022.

- EFSA regularly collects information on the presence of contaminants in food and feed, including data on the levels of MOH in food, from the EU Member States and other stakeholders. For the update of the risk assessment on MOH in food, EFSA considered occurrence data collected between 2011 and 2021.
- EFSA gathered past risk assessments from the websites of national and international organisations and targeted internet searches. Information on current legislation was obtained from EU institutions and the EU Member States' national legislations.

### What were the limitations/uncertainties?

- The large number of chemical compounds in the MOH group made it impossible to create a complete list of chemical characteristics.
- For MOSH, the Health-Based Guidance Value (HBGV) could not be established, so the Margin of Exposure (MoE) was used.
- For MOAH with 3- or more aromatic rings, there were not enough data on oral toxicity nor on their concentrations in food, to draw conclusions. However, the CONTAM Panel considered toxicity data from structurally similar substances (polycyclic aromatic hydrocarbons (PAHs)) and estimated exposure levels considering two possible occurrence scenarios to evaluate the risks.
- For MOAH with 1-2 rings, it was not possible to identify a dose at which a low but measurable adverse effect is observed (reference point), because there was insufficient data on oral toxicity.

### What were the outcomes and their implications?

The EC may take into account the following outcomes when considering whether to set maximum levels for MOH in certain foods:

- **It is likely to very likely (66-95% certain) that the present dietary exposure to MOSH does not raise concerns for human health.** Adverse effects on the liver previously observed in laboratory rats were concluded to be of no relevance for humans. Nevertheless, it is important to keep studying the possible long-term effects on human health.
- Additional data on toxicity and exposure are required for the final risk assessment of 3- or more aromatic ring MOAH. However, based on the above approach, **it is extremely likely (99-100% certain) for toddlers and likely (more than 66% certain) for other age groups that present dietary exposure to 3- or more aromatic ring MOAH, which are associated with DNA damage and potentially causing cancer, raises a possible concern for human health**.
- **Dietary exposure to 1-2 ring MOAH lacks reliable toxicity data, which might raise a concern**.
- The highest levels of MOH were found in vegetable oils and the population with the highest exposure was young people, mainly infants via infant formula.

### What are the key recommendations for public health authorities, policymakers and researchers?

**Recommendations for MOSH:**

- Determine concentrations in food as per the Joint Research Centre (JRC) guidance document (Bratinova and Hoekstra, 2023).
- Improve the analytical methodology for better MOSH characterisation and consistent reporting.
- Improve research on the sources of hydrocarbons in food.
- Collect data on the formation, fate and toxicity of products formed after MOSH is altered in the body, including their accumulation potential.
- Investigate the structural features of MOSH that inhibit human metabolism and elimination, leading to accumulation in the body.
- Collect additional data on the accumulation of MOSH in the body as well as on the toxic effects of MOSH, in particular in the liver, spleen, and immune and nervous systems.

- Research possible effects on lipoproteins, inflammation and inflammatory markers as seen in recent clinical trials.
- Collect additional data on MOSH concentrations in human tissue or develop and use biomarkers of exposure, especially for individuals born after 1995.
- Determine the contribution of environmental sources to the level of food contamination for MOSH in comparison to other sources of MOSH.

**Recommendations for MOAH:**

- Determine concentrations in food as per the JRC guidance document (Bratinova and Hoekstra, 2023).
- Collect more data on composition by aromatic ring number, mainly levels of 3- or more ring MOAH present in food.
- Collect more data on 3- or more ring MOAH and the influence of ring alkylation (transfer of an alkyl group) on damaging genes and causing cancer.
- Collect oral toxicity data for 1-2 ring MOAH.
- Investigate sources of food contamination when MOAH are detected.
- Update technical specifications with detailed information on the MOAH content and composition of white mineral oils and waxes used as food additives and food packaging materials.

### Glossary

Biomarker of exposure – a measurable substance in an organism whose presence is indicative of environmental exposure.

Health-Based Guidance Value (HBGV) – guidance on safe consumption of substances that takes into account current safety data, uncertainties in these data, and the likely duration of consumption.

Margin of Exposure (MoE) – a tool used in risk assessment to explore safety concerns arising from the presence of a potentially toxic substance in food or animal feed.

Mineral oil aromatic hydrocarbon (MOAH) – MOH containing one or more aromatic rings.

Mineral oil hydrocarbon (MOH) – a compound of hydrogen and carbon of mineral origin.

Mineral oil-saturated hydrocarbon (MOSH) – MOH containing only single bonds between carbon atoms.

Polycyclic aromatic hydrocarbons - a large class of organic compounds that are composed of two or more fused aromatic rings.

**Disclaimer**

- This plain language summary (PLS) is a simplified communication of EFSA's *Update of the risk assessment of mineral oil hydrocarbons (MOH) in food*.
- The full EFSA opinion can be found here: https://doi.org/10.2903/j.efsa.2023.8215.
- The purpose of the PLS is to enhance transparency and inform interested parties on EFSA's work on the topic using simplified language to present a summary of the main findings.
